# Characterization of an A-kinase anchoring protein-like suggests an alternative way of PKA anchoring in *Plasmodium falciparum*

**DOI:** 10.1186/s12936-016-1275-9

**Published:** 2016-04-29

**Authors:** Kossiwa Bandje, Bernina Naissant, Pascal Bigey, Murielle Lohezic, Marlène Vayssières, Magali Blaud, Laetitia Kermasson, José-Juan Lopez-Rubio, Gordon Langsley, Catherine Lavazec, Philippe Deloron, Anaïs Merckx

**Affiliations:** IRD UMR216-MERIT, Faculté de Pharmacie, Mère et enfant face aux Infections Tropicales, Paris, France; COMUE Sorbonne Paris Cité, Université Paris Descartes, Paris, France; Laboratoire d’Excellence GR-Ex, Paris, France; INSERM U1016, Institut Cochin, Paris, France; CNRS UMR 8104, Faculté de Médecine, Paris, France; Chimie ParisTech, PSL Research University, Paris, France; UMR CNRS 8015, Faculté de Pharmacie, Laboratoire de Cristallographie et RMN Biologiques, Paris, France; CNRS5290, IRD224, University Montpellier 1 & 2, MIVEGEC, Montpellier, France; Laboratoire d’Excellence ParaFrap, Paris, France; Institut Pasteur, Paris, France

**Keywords:** A‐kinase anchoring protein like (AKAL), 14-3-3 protein, *Plasmodium falciparum*, AMP, Interactome, Nucleotide

## Abstract

**Background:**

The asexual intra-erythrocytic multiplication of the malaria parasite *Plasmodium falciparum* is regulated by various molecular mechanisms. In eukaryotic cells, protein kinases are known to play key roles in cell cycle regulation and signaling pathways. The activity of cAMP-dependent protein kinase (PKA) depends on A-kinase anchoring proteins (AKAPs) through protein interactions. While several components of the cAMP dependent pathway—including the PKA catalytic and regulatory subunits—have been characterized in *P. falciparum*, whether AKAPs are involved in this pathway remains unclear. Here, PfAKAL, an open reading frame of a potential AKAP-like protein in the *P. falciparum* genome was identified, and its protein partners and putative cellular functions characterized.

**Methods:**

The expression of PfAKAL throughout the erythrocytic cycle of the 3D7 strain was assessed by RT-qPCR and the presence of the corresponding protein by immunofluorescence assays. In order to study physical interactions between PfAKAL and other proteins, pull down experiments were performed using a recombinant PfAKAL protein and parasite protein extracts, or with recombinant proteins. These interactions were also tested by combining biochemical and proteomic approaches. As phosphorylation could be involved in the regulation of protein complexes, both PfAKAL and Pf14-3-3I phosphorylation was studied using a radiolabel kinase activity assay. Finally, to identify a potential function of the protein, PfAKAL sequence was aligned and structurally modeled, revealing a conserved nucleotide-binding pocket; confirmed by qualitative nucleotide binding experiments.

**Results:**

PfAKAL is the first AKAP-like protein in *P. falciparum* to be identified, and shares 23 % sequence identity with the central domain of human AKAP18δ. PfAKAL is expressed in mature asexual stages, merozoites and gametocytes. In spite of homology to AKAP18, biochemical and immunochemical analyses demonstrated that PfAKAL does not interact directly with the *P. falciparum* PKA regulatory subunit (PfPKA-R), but instead binds and colocalizes with Pf14-3-3I, which in turn interacts with PfPKA-R. In vivo, these different interactions could be regulated by phosphorylation, as PfPKA-R and Pf14-3-3I, but not PfAKAL, are phosphorylated in vitro by PKA. Interestingly, PfAKAL binds nucleotides such as AMP and cAMP, suggesting that this protein may be involved in the AMP-activated protein kinase (AMPK) pathway, or associated with phosphodiesterase activities.

**Conclusion:**

PfAKAL is an atypical AKAP that shares common features with human AKAP18, such as nucleotides binding. The interaction of PfAKAL with PfPKA-R could be indirectly mediated through a join interaction with Pf14-3-3I. Therefore, PfPKA localization could not depend on PfAKAL, but rather involves other partners.

## Background

Malaria kills about 438,000 people per year, mostly in Africa [1]. *Plasmodium falciparum,* a protozoan parasite, is the deadliest of the five known species responsible for the disease in humans. Current malaria control strategies rely primarily on insecticide-treated bed nets and drugs. However, the extensive spread of drug resistance limits the armamentarium [2]. The identification and characterization of novel *Plasmodium*-specific proteins and their joint interaction may lead to the development of new therapeutic strategies.

Protein phosphorylation is one of the most common post-translational modifications involved in cellular communication. The phosphorylation status of a protein is determined by the balance between the activities of kinases and phosphatases. The cAMP-signalling pathway plays a central role in many developmental processes in eukaryotes, by activating molecules such as the cAMP-dependent protein kinase (PKA). A high concentration of cAMP interacting with regulatory subunits liberates catalytic subunits of PKA (PKA-C) to phosphorylate its target proteins. In eukaryotes, the subcellular specificity of PKA largely depends on its interaction with A-kinase anchoring proteins (AKAPs). This interaction ensures the spatio-temporal control of PKA activity, by tethering the enzyme close to its substrates, and by interacting with phosphatases and phosphodiesterases (PDE) [3–5].

AKAP proteins have a wide range of localizations, and can be associated with membranes, cellular organelles, and compartments. In the nucleus for instance, their localization is controlled through protein-lipid or protein–protein interactions [6]. The subcellular localization of AKAPs can be dynamic and regulated by phosphorylation [3]. Human AKAP18 is a low molecular weight AKAP with four different isoforms with distinct targeting domains. Because of their localizations, AKAP18 isoforms are involved in different cellular mechanisms. AKAP18δ, the larger isoform, has been reported to anchor PKA for both phosphorylation of aquaporin-2 in kidneys [4] and phosphorylation of phospholamban in the heart [5]. AKAP18δ also binds PDE4A isoforms within its central region [6]. A common central domain of AKAP18δ/γ has been described, whose structure resembles the 2H phosphoesterase family, harbouring a pair of conserved His-x-Thr motifs [7].

In *P. falciparum,* several effectors of the cAMP-pathways including cAMP-dependent protein kinase catalytic subunit (PKA-C) and regulatory subunit (PKA-R) homologues [8, 9], nucleotide cyclases [10] and phosphodiesterase [11, 12] have been identified. Importantly, the PfPKA-C knock-out is lethal and the PfPKA-R overexpression inhibits the growth of the parasite suggesting a crucial role of these effectors in the development and survival of the parasite [9].

In this study, the expression and function of the *P. falciparum* protein encoded by PF3D7_0512900/PFE0640w [PlasmoDB.org], herein named PfAKAL (*P. falciparum* A Kinase anchoring protein-like), based on its amino acid sequence homology with human AKAP18 [7], were investigated. However, unlike human AKAP18, PfPKA targeting and localization do not depend on direct binding to PfAKAL. This suggests that the subcellular localization of PfPKA may result from an alternative process and that the function of PfAKAL may differ from its human orthologs.

## Methods

### RNA extraction and real-time quantitative PCR experiments

*Plasmodium falciparum* 3D7 strain cultures were synchronized twice with 5 % d-sorbitol solution. The infected red blood cells were harvested every 8 h in order to have all the different intra-erythrocytic stages. Rings, early and late trophozoites, and schizont stages were harvested and conserved in Trizol. Stage-specific mRNA were extracted, and cDNA synthesized by RT-PCR. CGTAACGATGTTTTATAT (forward) and TAATATTTCTTTTGCGGG (reverse) primers specific to 140 pb of the *pfakal* gene were designed. Real-time Quantitative PCR was performed on the cDNA to estimate *pfakal* expression levels throughout the intra-erythrocytic development, using SensiFastSYBR NO-ROX Mix 2× (Bioline) and Rotor-gene™ 600 (Corbett Research), and 0.208 μM of each primer. The following PCR cycling conditions were used: initial heat activation step at 95 °C for 1 min, followed by 35 cycles of 95 °C for 15 s, 60 °C for 1 min and 72 °C for 5 min. All PCR reactions including negative controls (water) were carried out in duplicate, and a minimum of three experiments was carried out for each sample. The *p90* gene, encoding seryl-tRNA synthetase involved in DNA duplication was used as reference gene, and to characterize the RNA samples prior to the *pfakal* expression analysis. AAGTAGCAGGTCATCGTGGTT (forward) and TTCGGCACATTCTTCCATAA (reverse) primers specific to 158 pb of the *p90* gene were designed. The amplification specificity for each primer pair was determined by melting-curve analysis of each PCR product. The *pfakal* transcript abundance was calculated using the 2^−ΔΔCt^ method where Ct is the threshold cycle, and $\Delta \Delta {\text{Ct}}\,{ = }\left( {{\text{Ct}}\,pfakal{\mathbf{ - }}{\text{Ct}}\,p90} \right)_{{{\text{stage}}\,{\text{x}}}} {\mathbf{ - }}\left( {{\text{Ct}}\,pfakal{\mathbf{ - }}{\text{Ct}}\,p90} \right)_{{{\text{reference}}\,\,{\text{stage}}}}.$The result for each sample was expressed by calculating the mean and standard deviations between the three experiments. A statistical analysis was performed with a Kruskal–Wallis test.

### Molecular cloning and bacterial expression of recombinant proteins

Oligonucleotides were designed for amplification of a 798 bp region on chromosome 5, carrying the *pfakal* coding sequence and cloning into pGEX-6P1 (forward primer/*Bam*H1, cgGGATCCATGAATATTAAAAGAAGCATATACCA; reverse primer/*Xho*1, cgCTCGAGTCAAAGATTAAACTCAGACACG) *and* pRSF Duet (forward primer/*Bam*H1, GGATCCAATGAATATTAAAAGAAGCATATACCATTATTTAAG; reverse primer/*Not*1: GCGGCCGCTCAAAGATTAAACTCAGACACGATTT). Pf14-3-3I [PlasmoDB: MAL8P1.69/PF3D7_0818200] and PfPKA-R [PlasmoDB: PFL1110c/PF3D7_1223100] genes were also amplified by PCR prior cloning into pRSFDuet vector (forward *pf14*-*3*-*3I* primer/*Eco*RI: GAATCCGATGGCAACATCTGAAGAATT; reverse *pf14*-*3*-*3I* primer/*Not*1 GCGGCCGCTCATTCTAATCCTTCGTC; forward *pfpka*-*r* primer/*Eco*RI GAATTCGATGGGCAATGTGTGCAC; reverse *pfpka*-*r* primer/*Not*1 GCGGCCGCTTAATTTTCATCAATACAAGTTGTATCCA). After amplification from a *P. falciparum* cDNA library, the PCR products were digested prior insertion in the plasmids. For the different cloning steps, the inserts were verified by sequencing, and the plasmids were separately transformed into *Escherichia coli* BL21 Codon Plus (Stratagene) cells.

GST-PfAKAL, (His)6-PfPKA-R and (His)_6_-Pf14-3-3I protein expressions were induced for 4 and 6 h with 0.3 and 0.5 mM of Isopropyl β-D-1-thiogalactopyranoside (IPTG) at 37 °C, respectively. (His)_6_-PfAKAL expression was induced overnight with 0.3 mM of IPTG at 20 °C. All purification steps were performed at 4 °C. The bacterial pellets were lysed with lysosyme and by sonication in lysis buffer with ComplexTM mixture protease inhibitor tablet from Roche Applied Science. (GST-PfAKAL lysis buffer: 1× PBS, pH 7.5, 0.1 % Triton, 1 mM EDTA; (His)_6_-PfAKAL, (His)_6_-PF14-3-3I and (His)_6_-PfPKA-R lysis buffer: 20 mM Tris–HCl pH 8, 300 mM NaCl, 1 % Triton ×100). The lysates were cleared by centrifugation (9000 rpm at 4 °C for 1 h), and the soluble fraction was incubated for 1.5 h using mild agitation with glutathione-agarose (Sigma-Aldrich) or Ni–NTA-agarose (Invitrogen) resin.

GST-PfAKAL slurry was washed in lysis buffer, and the fusion protein eluted with elution buffer (30 mM Tris, pH 8.0, 15 mM NaCl, 20 mM glutathione). (His)_6_ tagged protein slurry was washed three times in lysis buffer, twice in lysis buffer with 20 mM imidazole, and eluted with elution buffer (lysis buffer with 250 mM Imidazole). All recombinant protein preparations were confirmed by anti-GST or anti-(His)_6_ western blot analysis.

### Gel filtration analysis

To determine if proteins are forming dimer, gel filtration analysis were performed on the eluted fractions obtained after affinity purification of (His)_6_-Pf14-3-3I and (His)_6_-PfPKA-R. Samples were loaded onto a Superdex S200 10/300 Increase column (GE Healthcare) and gel filtration were carried out using GF buffer (20 mM Tris–HCl at pH 8, 300 mM NaCl). The fractions corresponding to the pic detected by UV were analysed by a 12.5 % SDS-PAGE to confirm the size of the eluted proteins.

### Immunofluorescence assay (IFA)

In vivo immunizations were carried out on 6-week old Swiss female mice (Janvier, Le Genest De L’Isle, France). Four mice were first immunized by an intraperitoneal injection of 10 µg of recombinant PfAKAL fused to a GST tag (produced as described above) homogenized in 1 mg aluminium hydroxide (Alugel, Serva) in a 100 µl saline volume. Two boosts of 10 µg of recombinant PfAKAL fused to a (His)_6-_tag homogenized in 1 mg aluminium hydroxide in a 100 µl saline volume were administered on days 21 and 42. Blood samples were collected by retro-orbital bleeding at day 56. Rat Anti Pf14-3-3I antibodies were obtained according to [13].

*Plasmodium falciparum* 3D7 strain infected red blood cells with 7 % parasitaemia (mostly trophozoites and schizonts) were washed in PBS. The cells were fixed with methanol. After overnight incubation in PBS, 2 % BSA the blood smears were incubated with one of the following antisera: anti-PfAKAL mouse antiserum diluted 1:500 and anti-Pf14-3-3I rat antibodies diluted 1:100, or an anti-PfAMA1/28G2d described in [14] and diluted 1/1.000 (kindly provided by J.C. Barale, Institut Pasteur, Paris). After washes, the slides were incubated with AlexaFluor conjugated secondary antibodies against mouse or rat IgG containing 1/20,000 Hoechst (Hoechst 33,342, trihydrochloride, trihydrate 10 mg⁄mL) and mounted in vectashield (Vectorslab). Samples were observed at 100× magnification using an Olympus fluorescent microscope. Thereafter, the PfAKAL and Pf14-3-3I cellular localizations were analysed using the Imaris deconvolution software from Bitplane.

### Peptide array experiment

128 overlapping 15 mer-peptides with a two amino acids frameshift, derived from the full-length PfAKAL amino acid sequence were synthesized on nitrocellulose membrane. The membrane was incubated in a 1 µM (His)_6_-PfPKA-R protein recombinant solution. An anti-polyhistidine western blot was performed to identify the peptides having interacted with (His)_6_-PfPKA-R.

### Pull-down experiments with parasite protein extracts

*Plasmodium falciparum* (3D7) pellets were sonicated in RIPA buffer (30 mM Tris, pH 8.0, 150 mM NaCl, 20 mM MgCl_2_, 1 mM EDTA, 1 mM dithiothreitol, 10 µM ATP, 0.5 % Triton X-100, 1 % Nonidet P-40, Complete EDTA free protease inhibitors Coktail (Roche), and PhosSTOP Phosphatase inhibitors Coktail (Roche). The lysates were cleared by centrifugation (15,000 rpm for 30 min at 4 °C), and the total amount of protein in the supernatant was measured using a Coomassie Plus Protein Assay Reagent (Pierce). The beads coated with GST, GST-PfAKAL or AMP were incubated with the parasite extracts or in RIPA buffer alone at 4 °C, under mild agitation for 90 min (100 µg of total parasite proteins for 10 µg of recombinant protein on beads). The beads were then washed three times in RIPA buffer, and once in RIPA buffer with 0.1 % SDS. The precipitated complexes were analysed by mass spectrometry (Proteomic Facility, 3P5 Université Paris Descartes).

### Protein/protein and protein/nucleotide interaction experiments

GST, GST-PfAKAL glutathione beads were incubated with (His)_6_-Pf14-3-3I or (His)_6_-PfPKA-R recombinant proteins. Also, GST and GST-PfPKA-R glutathione beads were incubated in (His)_6_-Pf14-3-3I recombinant protein. Adenosine monophosphate (AMP) or cyclic AMP cross-linked agarose beads were incubated in recombinant (His)_6_-PfAKAL protein solution for 2 h at 4 °C.

For all pull down experiments, beads were washed in RIPA buffer, and the protein complexes were analysed by SDS-PAGE and an anti-histidine western blot (Sigma Aldrich Monoclonal Anti-polyHistidine Peroxidase Conjugate) was performed to identify interactions.

### Kinase assay

Assays were performed in a standard reaction (30 µl) containing 50 mM MOPS pH7, 0.5 mM MgCl_2_, 15 µM ATP, 5 µCi of [γ-32P]ATP (3000 Ci/mmol; Perkin Elmer) and 1 µM of recombinant (His)_6_-PfPKA-R, 1 µM recombinant (His)_6_-Pf14-3-3I or 1 µM recombinant (His)_6_-PfAKAL. The reactions were initiated by addition of 1 µM commercial bovine PKAc (Sigma Aldrich P2645), with or without 1 µM H89, a potent PKA inhibitor. The kinase reactions, proceeded for 30 min at 30 °C, were stopped by the addition of Laemmli buffer, boiled for 3 min and analysed by electrophoresis on 8 % SDS–polyacrylamide gel. The gels were dried and submitted to autoradiography.

### PfAKAL structural model and binding site predictions

Sequence alignments of PfAKAL to *Plasmodium yoelii* AKAP18 [PlasmoDB: PY04627], a *Plasmodium vivax* ortholog [PlasmoDB: PVX080595] and human AKAP18 γ isoform were performed using ClustalO, and visualized using BOXSHADE. Amino acid sequences of PfAKAL was submitted to the I-TASSER server for structural prediction [15, 16]. Protein structures were visualized using PyMol version 0.99rc6.

## Results and discussion

### In silico identification of a *P. falciparum* ORF related to an AKAP

**Fig. 1:**
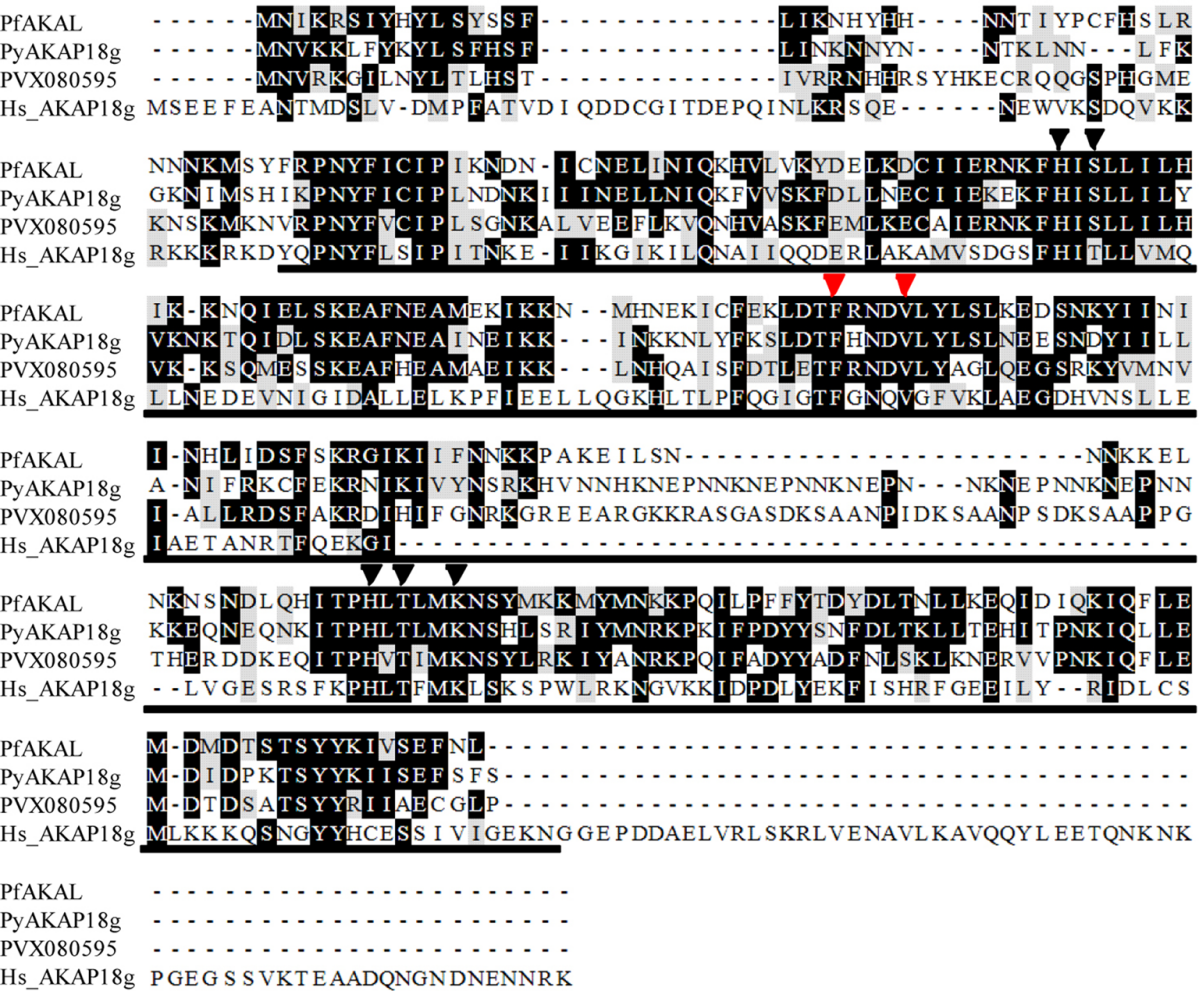
Alignments of PfAKAL, *P. yoelii* AKAP18 (PyAKAP18g) and *P. vivax* ortholog (PVX080595) with Human AKAP18γ. In this *box shade* representation of the alignment, identical and similar residues are shaded respectively in *black* and *grey*. The residues involved in nucleotide binding are labeled with* triangles* above the alignment, the conserved residues interacting with the adenosine (*red*) and the residues interacting with the phosphate (*black*) moieties of 5′AMP in the human AKAP18 complex crystal structure. The human AKAP18 core domain is underlined with plain line

BLASTP searches of the PlasmoDB database were performed, using as queries a variety of AKAP sequences from different organisms, identifying a single *P. falciparum* polypeptide displaying significant amino acid homology. The predicted 265-residue protein, PfAKAL [PlasmoDB PFE0640w/PF3D7_0512900] displays 23 % identity, and 33 % similarity to human AKAP18γ. The parasite protein also shows homology to predicted proteins from other *Plasmodium* species, such as 50 and 44 % identities with *P. yoelii* [PlasmoDB: PY04627], and *P. vivax* [PlasmoDB: PVX080595], respectively (Fig. 1). However, PfAKAL does not share all of the characteristics typical of mammalian AKAPs, such as, for example, a transmembrane domain, N-myristoylation, or palmytoylation sites suggesting that PfAKAL is cytosolic as are human AKAP18δ and AKAP18γ [17]. In addition to its homology with the core domain of human AKAP18γ, PfAKAL contains a Pfam AKAP7 2′5′ RNA ligase-like domain (pfam10469). This domain corresponds to the N-terminal part of AKAP7 which is known to play a role in regulating PKA-mediated gene transcription in somatic cells and oocytes [17]. PfAKAL also harbours two His-x-Thr/Ser motifs (Fig. 1) that define the 2H phosphoesterase family, including RNA ligases and cyclic nucleotide phosphoesterases [18]. Such residues are conserved in the AKAP18δ/γ from different organisms [7]. Despite the weak amino acid homology, PfAKAL is the only AKAP-like protein to be identified in the predicted proteome of *P. falciparum* and its role remains unknown.

### PfAKAL expression and localization throughout the erythrocytic stage

**Fig. 2:**
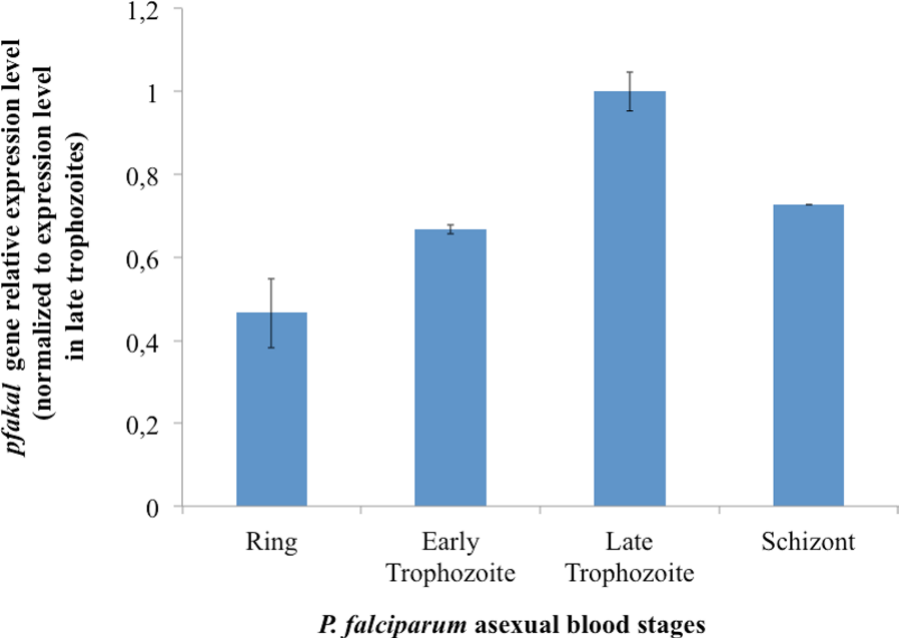
*Pfakal* expression levels in infected red blood cells. RT-qPCR analyses were performed on synchronized *P. falciparum* asexual blood stages. The *pfakal* transcript abundance was calculated using the 2^−ΔΔCt^ method where Ct is the threshold cycle, and $\Delta \Delta {\text{Ct}}\,{ = }\left( {{\text{Ct}}\,pfakal{\mathbf{ - }}{\text{Ct}}\,p90} \right)_{{{\text{stage}}\,{\text{x}}}} {\mathbf{ - }}\left( {{\text{Ct}}\,pfakal{\mathbf{ - }}{\text{Ct}}\,p90} \right)_{{{\text{late}}\,{\text{trophozoite}}}}$. Expression levels were normalized to *pfakal* gene transcription level in late trophozoites, the maximal expression observed throughout the erythrocyte cycle. The result for each sample was expressed by calculating the mean and standard deviations (*bars* on *histograms*) between the three experiments. A statistical analysis was performed with a Kruskal–Wallis test (p = 0.0077)

To determine whether *pfakal* is expressed during red blood cell infection, real-time quantitative PCR (RT-qPCR) experiments were performed on total cDNA from synchronized parasite erythrocytic stages from in vitro cultures of the 3D7 strain [19]. Primers efficiency were validated at 1.98 and 1.93 for *p90* and *pfakal* gene respectively, and a Kruskal–Wallis test was performed on the results, with a *p* value = 0.0077. The *pfakal* gene is expressed throughout the erythrocytic cycle, with maximal expression in mature trophozoite stages (Fig. 2), thus the expression levels were normalized to *pfakal* gene transcription level in late trophozoites (reference stage in ΔΔCt equation). The results correspond with those previously described in a large scale study [20].

**Fig. 3:**
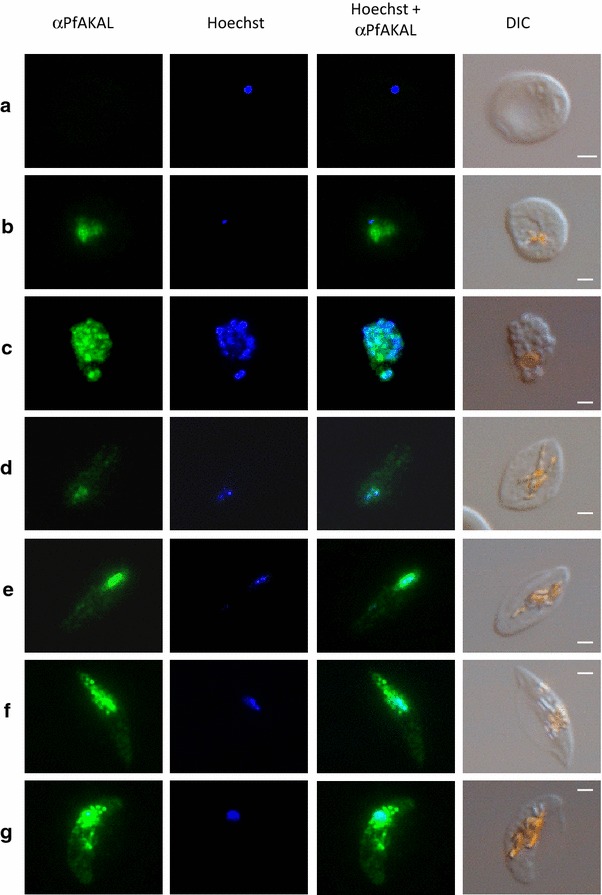
PfAKAL subcellular localizations in asexual and sexual *P. falciparum* blood stages. Images with PfAKAL localization (*green*), Hoechst DNA staining (*blue*), differential interference contrast (DIC) were taken on rings **a** trophozoites, **b** schizonts, **c** as well as on stage II, **d** stage III, **e** stage IV, **f** and stage V, **g** gametocytes, infected erythrocytes. “*Bright orange*” on DIC images indicates haemozoin crystals. The *bars* represent 2 μm

**Fig. 4:**
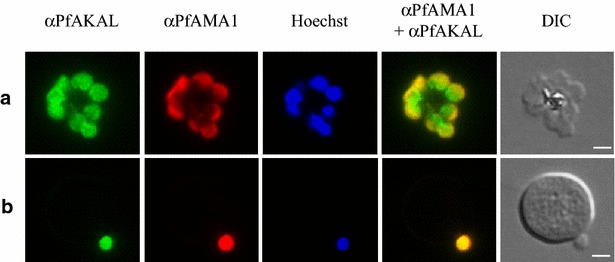
PfAKAL localization in *P. falciparum* merozoites by indirect immunofluorescence assays. Images with PfAKAL localization (*green*), PfAMA1 localization (*red*), Hoechst DNA staining (*blue*) and differential interference contrast (DIC) and were taken. **a** *P. falciparum* 3D7 egressing merozoites, **b** *P. falciparum* 3D7 invading merozoite. The *bars* represent 2 μm

To analyse the subcellular distribution and stage specificity of PfAKAL expression, immunofluorescence assays were carried out on *P. falciparum*-infected erythrocytes harbouring parasites at different development stages (Fig. 3). In asexual parasites PfAKAL was undetectable in ring stages (Fig. 3a), with expression predominantly in late trophozoites and schizonts. Interestingly, a diffuse subcellular localization of PfAKAL was observed in the parasite cytoplasm and in the nucleus in trophozoite stages, whereas it was mostly nuclear in schizonts (Fig. 3b, c). In sexual stages, PfAKAL was also detected in the cytoplasm of the parasite, although it mainly accumulates around the nucleus (Fig. 3d–g). To analyse the PfAKAL localization on merozoïtes immunofluorescence assays were also performed with a dual labelling using mouse anti-PfAKAL antibody with a rat anti-PfAMA1, a control labelling parasite plasma membrane. PfAKAL shares partial location with PfAMA1, and the IFA results showed that PfAKAL is expressed in egressing (Fig. 4a) and invading (Fig. 4b) merozoites, in agreement with a wide proteomic analysis of *P. falciparum* cycle [21].

These results strongly suggest that PfAKAL is expressed at the transcript level as well at the protein level during the intra-erythrocytic development of *P. falciparum* parasites, but PfAKAL remains within the parasite and is not exported to the infected red blood cell cytosol. The data is consistent with the in silico analysis suggesting that PfAKAL is not a membrane associated protein.

### PfPKA anchoring does not depend on direct interaction with PfAKAL

**Fig. 5:**
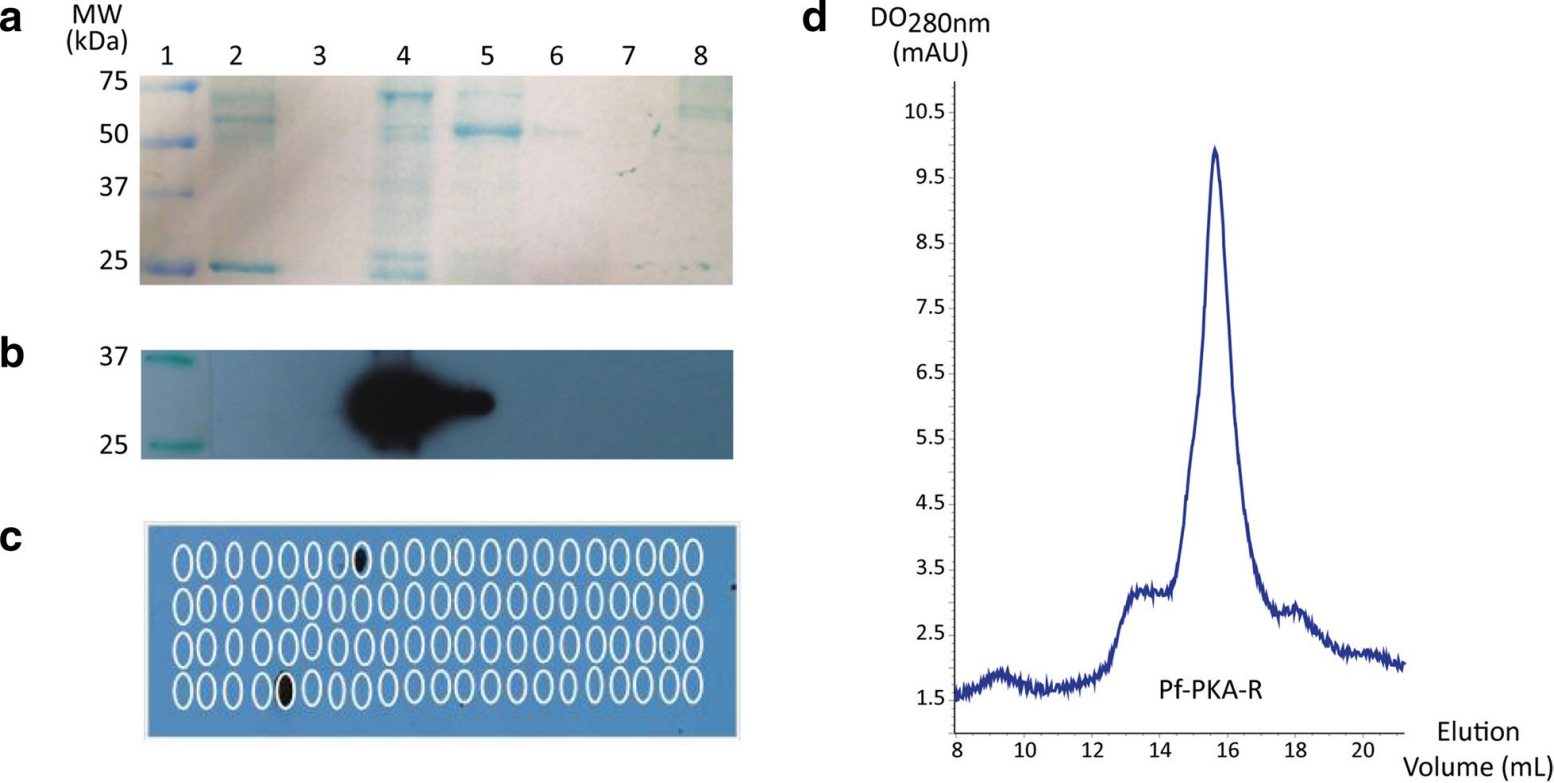
Interaction between GST-PfPKA-R and (His)_6_-PfAKAL protein. **a** Nitrocellulose membrane coloration and anti-polyhistidine Western blot (**b**) after GST beads (2) and GST-PfPKA-R beads (8) were incubated in (His)_6_-PfAKAL solution. Molecular marker (1), empty lane (3), (His)_6_-PfAKAL (4), flowthrough after incubation of GST-PfPKA-R beads in (His)_6_-PfAKAL solution (5), beads washes (6–7). The recombinant GST-PfPKA-R and (His)_6_-PfAKAL proteins molecular weight are 75 and 32 kDa respectively. **c** A peptide array was performed using peptides derived from the PfAKAL whole protein sequence. An anti-polyhistidine western blot was realized to identify the peptides interacting with (His)_6_-PfPKA-R. **d** Gel filtration analysis of (His)_6_-PfPKA-R

In human cells, AKAPs bind PKA through an amphipathic α-helical structure consisting of 14–18 amino acids (RII-binding domain), which interacts with the hydrophobic groove formed by the N-terminal dimerization and docking (D/D) domain of PKA-R subunits [22]. To investigate the potential interaction between PfAKAL and PfPKA regulatory subunit, recombinant proteins were produced and used for GST pull-down assays. Purified GST-PfPKA-R beads were incubated in purified (His)_6_-PfAKAL solution. Anti-polyhistidine western blot analysis failed to reveal any interaction between the two proteins, suggesting that there is no direct interaction between the two proteins (Fig. 5a, b, lane 8). The ability of PfAKAL to bind PfPKA-R was also investigated using a peptide array analysis. To this end, recombinant (His)_6_-PfPKA-R was incubated with a membrane containing PfAKAL derived peptides and again, anti-polyhistidine immunoblot analysis did not reveal the presence of (His)_6_-PfPKA-R on the membrane. Thus, this particular array did not reveal any interaction between the two proteins. The two positive dots correspond to a positive control peptide (containing six histidines, bottom line) and a derived peptide from PfAKAL containing three histidines (top line) (Fig. 5c).

Even though peptide array technology has been used successfully to study PKA-R/AKAP interaction in other organisms [23, 24], the fact that a 15-mer peptide doesn’t mimic the complete docking site cannot be excluded, nor the possibility of peptide and PfAKAL or PfPKA-R recombinant protein mis-folding.

Therefore, to determine if PfPKA might be an interacting partner of PfAKAL in the parasite, pull-down experiments using recombinant GST-PfAKAL and native parasite protein extracts were performed and followed by mass spectrometry nLC-MSMS analysis. This approach confirmed the absence of any direct interaction between PfAKAL and PfPKA-R.

Furthermore, an In silico analysis of PfPKA-R highlighted a difference of the N-terminal portion compared to the equivalent N-terminal part of human PKA-R, as it does not share the prototypical helical bundle that allows dimerization of PKA-R subunits, and the docking of AKAP proteins [25]. An analytical gel filtration analysis on (His)_6_-PfPKA-R was also performed, and showed only one pic (Fig. 5d). This pic corresponds to a monomer of (His)_6_-PfPKA-R, according to the calibration of the Superdex S200 10/300 Increase column (GE Healthcare) around a molecular weight of 50 kDa. The eluted fractions from the pic were collected and analysed by 12.5 % SDS-PAGE to confirm that the pic corresponds to (His)_6_-PfPKA-R. These observations are in agreement with the lack of direct interaction between PfAKAL and PfPKA-R, and raise the hypothesis of an alternative way of PKA anchoring in the malaria parasite. This hypothesis was recently reinforced by the antimalarial activity of a human AKAP disruptor peptide STAD-2, through a PKA-independent mechanism [26], and by a recent analysis highlighting the fact that AKAP can only be found in animal genomes, and likely evolved in conjunction with multicellularity [27]. Several PKA phosphorylation sites have however been identified in the N-terminal part of PfPKA-R [28–30], and Haste and colleagues suggested that these sites might be involved in the docking of other proteins such as Pf14-3-3 [25].

### PfAKAL/PfPKA complex might depend on Pf14-3-3I

**Fig. 6:**
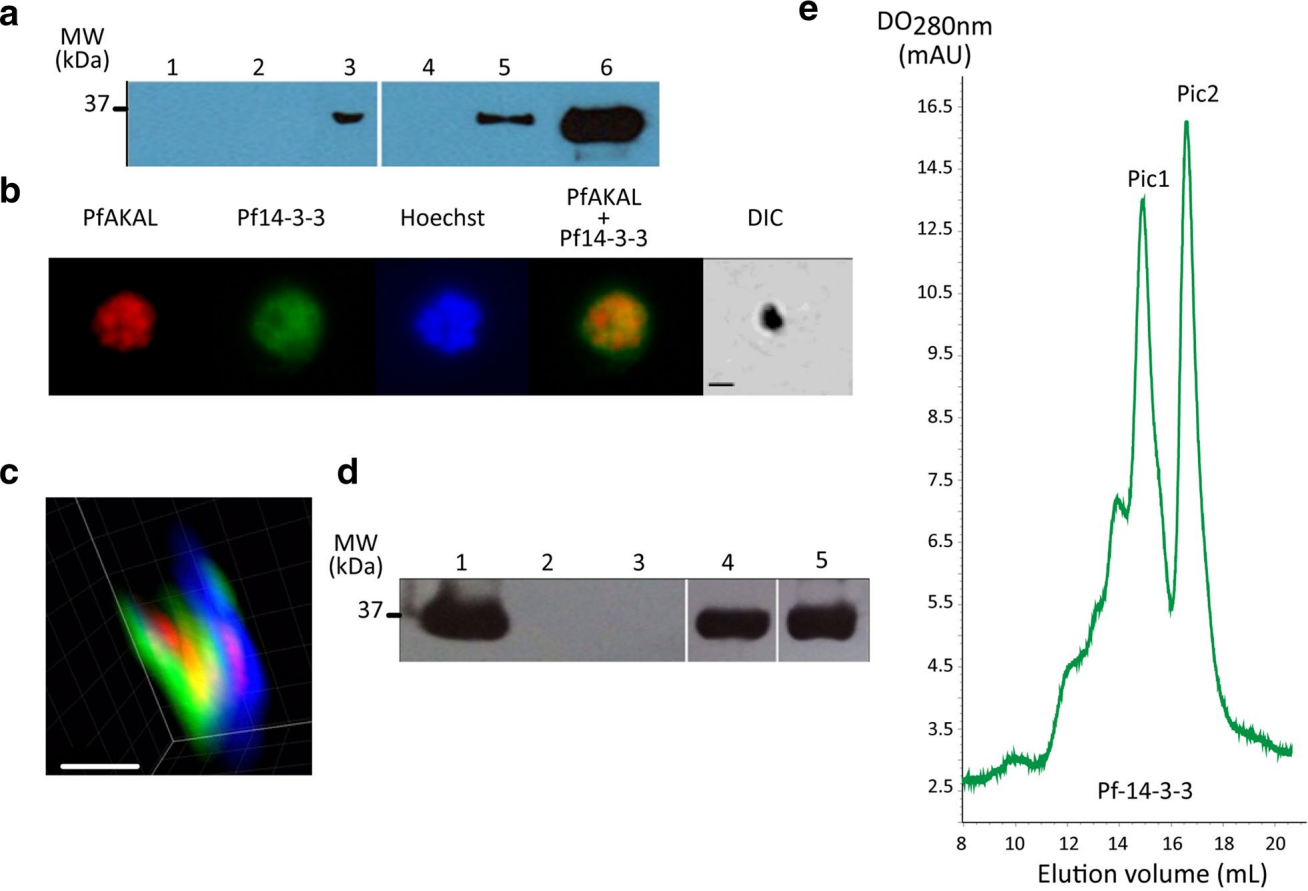
(His)_6-_Pf14-3-3I interaction with GST-PfAKAL and GST-PfPKA-R. **a** Anti-polyhistidine western blot analysis after incubation of GST-PfAKAL beads in (His)_6_-Pf14-3-3I solution. GST beads (1), molecular marker (2), flowthrough after incubation of GST-PfAKAL beads in the (His)_6_-Pf14-3-3I solution (3), glutathione agarose bead (4), and GST-PfAKAL beads (5), incubated in (His)_6_-Pf14-3-3I solution, (His)_6_-Pf14-3-3I (6). **b** PfAKAL and Pf14-3-3I localizations by immuno-staining of 3D7 parasites infected erythrocytes smears with mouse anti-PfAKAL (*red*) and rat anti-Pf14-3-3I (*green*) antibodies. The merge shows colocalization of the two proteins (*yellow*). **c** Deconvolution microscopy analysis (Imaris software) on a trophozoite parasite showing PfAKAL (*red*) and Pf14-3-3I (*green*) localizations in the infected red blood cell, nucleus staining (blue). The *pink* and *yellow* colors represent the localizations where *blue* and *red* signals or *green* and *red* signals are merged respectively. The *bars* represent 2 μm, **d** Anti-polyhistidine western blot analysis after incubation of GST-PfPKA-R beads in (His)_6_-Pf14-3-3I solution. (His)_6_-Pf14-3-3I (1); GST beads in (His)_6_-Pf14-3-3I solution (2); molecular marker (3); GST-PfAKAL beads in (His)_6_-Pf14-3-3I solution (4); GST-PfPKA-R beads in (His)_6_-Pf14-3-3I solution (5). **e** Gel filtration analysis of (His)_6_-Pf14-3-3I showing two pics: pic 1 corresponds to a dimer of (His)_6_-Pf14-3-3I and pic2 corresponding to a monomer of (His)_6_-Pf14-3-3I

The combination of pull-down experiments with GST-PfAKAL beads incubated with parasite protein extracts and nLC-MSMS analysis identified Pf14-3-3I [PlasmoDB: MAL8P1.69/PF3D7_0818200] [13], as a putative PfAKAL partner. We further investigated this interaction with GST-PfAKAL and (His)_6_-Pf14-3-3I recombinant proteins. Purified GST-PfAKAL beads were incubated in purified (His)_6_-Pf14-3-3I solution, and binding analysed by anti-polyhistidine western blot. As expected, GST-PfAKAL bound (His)_6_-Pf14-3-3I, while GST alone did not (Fig. 6a). To confirm this interaction, co-immunofluorescence assays were performed on infected red blood cells with anti-PfAKAL and anti-Pf14-3-3I antibodies. Pf14-3-3I is known to be present in both cytoplasmic and nuclear compartments of the parasite [13], but these locations seem more diffuse than that of PfAKAL throughout the parasite development. The distribution of the two proteins partially overlapped in the parasite cytoplasm and nucleus (Fig. 6b). Partial co-localization was confirmed following deconvolution of the IFA images (Fig. 6c). When combined with pull-downs, the IFA images confirm the interaction between PfAKAL and Pf14-3-3I.

14-3-3 proteins are known to play scaffold functions in cells to stabilize multiple enzyme complexes including PKA subunits [31], and to be phosphorylated by cAMP-dependent kinases [32]. Therefore the interaction between Pf14-3-3I and PfPKA-R was investigated by pull-down experiments involving recombinant proteins. Purified GST-PfAKAL, GST-PfPKA-R and GST (as control) beads were incubated in purified (His)_6_-Pf14-3-3I solution, and the complexes analysed by anti-polyhistidine western blot (Fig. 6d). Both PfAKAL (Fig. 6d, lane 4) and PfPKA-R (Fig. 6d, lane 5) interact with Pf14-3-3I suggesting that Pf14-3-3I could mediate binding between the two proteins acting as a scaffold in the assembly of a PfPKA/PfAKAL complex. As PfPKA-R binds Pf14-3-3I it also suggests that Pf14-3-3I might be phosphorylated by PfPKA-C, as phosphorylation of 14-3-3 by PKA has been reported to play a role in the regulation of the dimerization of 14-3-3 [32]. An analytical gel filtration performed on (His)_6_-Pf14-3-3I samples demonstrated the dimerization of Pf14-3-3I, as two pics were obtained on Superdex S200 10/300 Increase column (GE Healtcare), corresponding to the size of a dimer (around 62 kDa) and a monomer of (His)_6_-Pf14-3-3I (around 31 kDa) (Fig. 6d). The nature of the proteins in the two eluted fractions was confirmed by 12.5 % SDS-PAGE analysis.

### PKA phosphorylates PfPKA-R and Pf14-3-3I

**Fig. 7:**
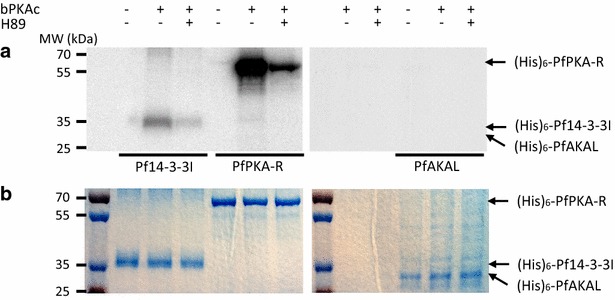
Phosphorylation of (His)_6_-PfPKA-R, (His)_6_-Pf14-3-3I or (His)_6_-PfAKAL by bovine PKA catalytic subunit (bPKA). Radiolabel kinase activity assays were deployed to detect phosphorylation of the proteins. Assays without PKA or with H89, a potent PKA inhibitor were used as negative controls. Coomassie blue staining (**b**) of radiolabelled SDS-gels (**a**) was used as a loading control

First, the PfAKAL protein sequence was analysed with an algorithm predicting PKA phosphorylation potential sites [33] and identified a single site with a very weak score. To investigate the potential phosphorylation of PfPKA-R and Pf14-3-3I, an in vitro phosphorylation assay was performed with recombinant proteins (His)_6_-Pf14-3-3I, (His)_6_-PfPKA-R and (His)_6_-PfAKAL. *Plasmodium falciparum* (PfPKA-C) and bovine PKA catalytic subunits (PKA-C) share 48 % similarity in the overall primary sequence, but 80 % similarity in the activation segment that interacts with the substrate [34]. PKA phosphorylation sites also share a consensus sequence, R–R-X-S/T-Φ, where Φ represents a hydrophobic residue. Therefore bovine PKA catalytic subunit could be used in our kinase assays, to assess the phosphorylation of the different proteins. The results showed the phosphorylation of PfPKA-R and Pf14-3-3I by bovine PKA, and that these phosphorylations are inhibited by the potent PKA inhibitor H89 (Fig. 7). On the contrary, PfAKAL was not phosphorylated in vitro by bovine PKA (Fig. 7). The results confirmed the prediction of a weak probability for PfAKAL to be phosphorylated by PKA. Also, the PKA phosphorylation of PfPKA-R and Pf14-3-3I were reinforced by *P. falciparum* schizont phosphoproteome studies [29, 30].

As 14-3-3 proteins act as chaperones and bind actin to promote nuclear import or export [35, 36], Pf14-3-3I could play a role in the localization of PfAKAL, depending on the PKA-dependent phosphorylation state of Pf14-3-3I. This hypothesis needs to be further investigated.

### In silico structural analysis of PfAKAL protein reveals conservation of residues responsible for nucleotide binding

**Fig. 8:**
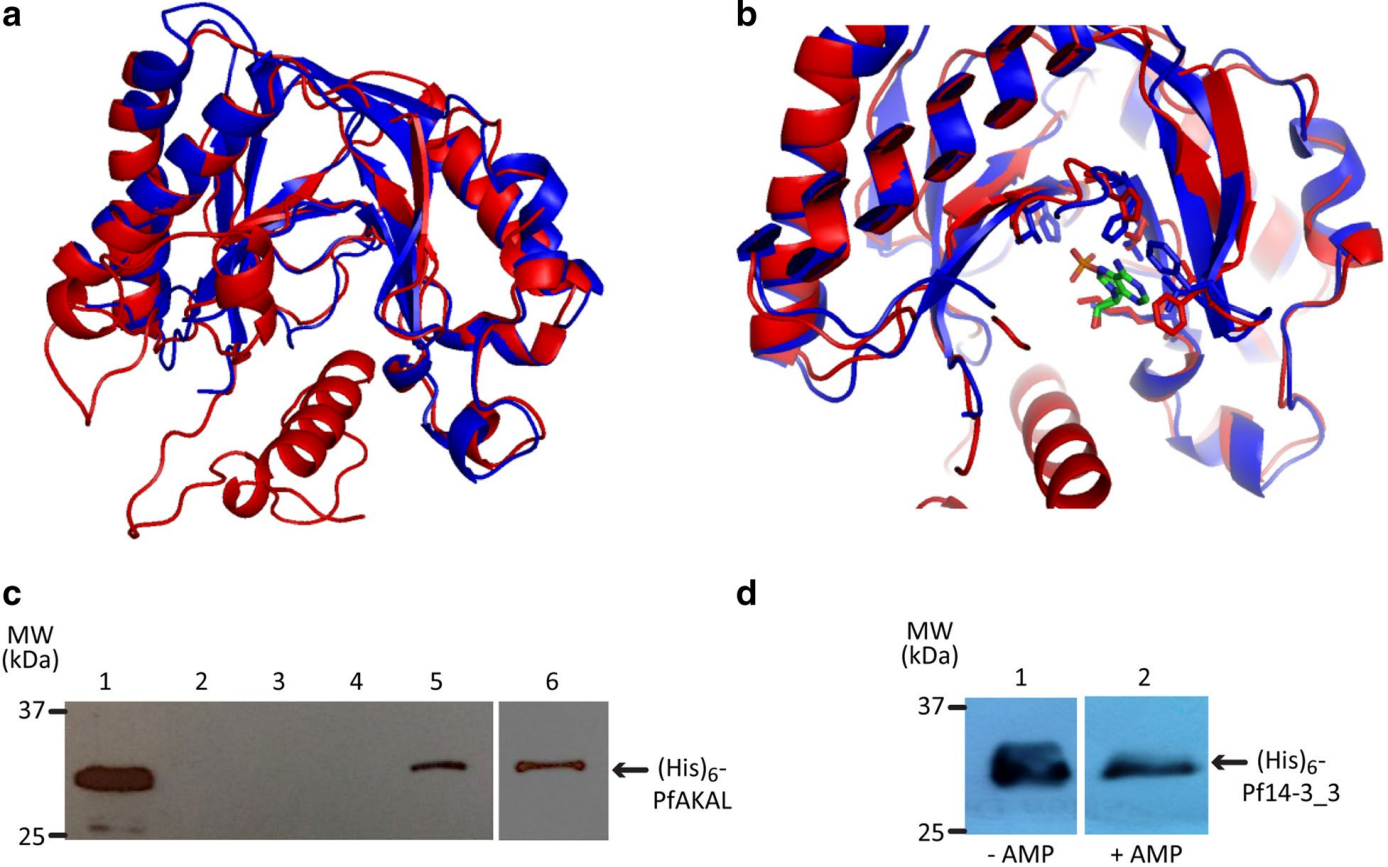
PfAKAL homology-based structural model and nucleotide binding studies, **a** Superposition of PfAKAL model with human AKAP18 central domain [PDB: 2vfl], represented with ribbon diagrams colored respectively in *red* and *blue*. **b** Superposition of PfAKAL model (*red*) with human AKAP18 that binds AMP [PDB: 2vfk] (*blue*). The residues involved in nucleotides binding are displayed as sticks, and AMP is colored in *green*. **c** Anti-polyhistidine western blot performed on precipitated protein complexes using cAMP- and AMP- agarose beads in (His)_6_-PfAKAL solution. (His)_6_-PfAKAL (1); beads washes (2–4), cAMP beads in (His)_6_-PfAKAL solution (5), AMP beads in (His)_6_-PfAKAL solution (6). **d** Anti-polyhistidine western blot analysis after incubation of GST-PfAKAL beads in (His)_6_-Pf14-3-3I solution in the presence (1) or absence (2) of 5 mM 3′–5′AMP; recombinants (His)_6_-PfAKAL and (His)_6_-Pf14-3-3I proteins molecular weight are 32 and 30 kDa respectively

To determine the potential function of PfAKAL, the amino acid sequence was submitted to the I-TASSER server for protein structure prediction [15, 16]. The returned sequence alignments and structural analogues were human AKAP18, bacterial 2′–5′ RNA-ligase, cyclic nucleotide phosphodiesterase from *Arabidopsis thaliana*. The I-TASSER server also predicted five structural models for PfAKAL. The backbone chains of all five structural models share the same general shape, but differ in some secondary structures. In the highest scoring model, the N-terminus is folded in an alpha-helix, but its conformation and location are different from that observed in the other models, and a binding pocket is present. The back of this pocket is lined by two β-sheets, whereas there is only one β-sheet in the other models (Fig. 8a). This highest scoring model is displayed alongside the structure of human AKAP18 co-crystallized with cytidine-5′-monophosphate (5′CMP) [7]. A superposition of PfAKAL structural model and human AKAP18 core domain structure confirmed that the two proteins share a similar shape (Fig. 8a). I-TASSER also predicted some binding sites [37] using human AKAP18 core domain and two phosphodiesterases from *Arabidopsis thaliana* and mouse. The highest scoring model, containing the predicted binding site from human AKAP18, highlights a possible interaction between PfAKAL and nucleotides such as AMP. The residues involved in AMP binding are conserved, but with some modifications in the orientation of the side chains (Fig. 8b).

A previous study also suggested that human AKAP18 acts as an adenosine monophosphate (AMP) sensor in the cell, binding AMP within a groove, located between the two lobes of the protein, and specifically binding 5′AMP and 5′CMP [7]. To investigate the nucleotide-binding capability of PfAKAL with 5′AMP and 3′–5′ cAMP, pull-down experiments were performed with 5′AMP immobilized on agarose beads in a solution of recombinant PfAKAL or in parasite protein extracts. Western blot analyses showed that recombinant (His)_6_-PfAKAL interacts with 5′-AMP (Fig. 8c).

A mass spectrometry analysis of the native complexes from parasite protein extracts interacting with AMP-beads also confirmed that native PfAKAL is a 5′-AMP binder. The binding of 5′-AMP did not interfere with the interaction between PfAKAL and Pf14-3-3I (Fig. 8d). AMP binding by PfAKAL suggests different functions for the protein. One possibility is that PfAKAL acts as a 5′-AMP effector within the parasite, where classically 5′-AMP acts as an energy sensor triggering the AMP-activated protein kinase (AMPK) signalling pathway. In eukaryotic cells, AMPK is ubiquitous and acts as a cellular energy sensor, monitoring the AMP:ATP ratio. If an energy deficit is detected, AMPK activates or inhibits other pathways to restore energy homeostasis [38]. An AMPK has not been identified in *P. falciparum* until now, but BLAST analysis at PlasmoDB identified a putative actor in the AMPK signalling pathway, PfKIN (PlasmoDB identifier PF3D7_1454300), an as yet uncharacterized protein. Hanson and colleagues have shown that torins, a single structural class of mTOR inhibitors, are potent anti-malarials despite the absence of a recognizable mTOR kinase in *Plasmodium*. As mTOR kinases are AMPK downstream targets [39], this could suggest the presence of an AMPK signalling pathway in malaria parasites [40]. Thus, a third hypothesis is that PfAKAL acts as an AMP-binder and a scaffolding protein in this putative pathway. Therefore, the interaction of recombinant (His)_6_-PfAKAL with 3′–5′ cAMP was investigated (Fig. 8c). But the ability of PfAKAL to hydrolyse cAMP, because of its homology with bacterial phosphoesterases, still need to be established. Different AKAPs such as human AKAP18δ and mAKAP, have been shown to interact with PDE [41, 42], and PDE isoforms have been characterized in *Plasmodium* [11, 12] rendering possible an interaction between PfAKAL and PfPDEs.

## Conclusions

This study presents the characterization of PfAKAL, a protein initially identified as a putative PfPKA anchor. Using molecular and biochemical approaches, we have demonstrated that PfAKAL is expressed in all intraerythrocytic stages, including merozoites and gametocytes, but could not establish any direct protein–protein interaction with PfPKA-R. This suggests an alternative way of anchoring PfPKA within the parasite, and Pf14-3-3I is a PfPKA-R binding partner. The dimerization of Pf14-3-3I proteins could stabilize the N-terminus of PfPKA-R promoting the dimerization of PfPKA-R monomers, so creating a docking domain for yet to be identified parasite AKAPs. As Pf14-3-3I is PKA phosphorylated by bovine PKA in vitro, Pf14-3-3I dimerization and/or Pf14-3-3I interaction with PfPKA-R In vivo may be regulated by phosphorylation. The results presented here also highlight structural and biochemical similarities between PfAKAL and human AKAP18γ, including nucleotide binding such as AMP and cAMP. PfAKAL may play a role as an AMP sensor, or be involved in other pathways, such as AMPK or PDE pathways.

## Abbreviations

AKAP: a-kinase anchoring protein
AKAL: a kinase anchoring protein-like
AMP: adenosine monophosphate
cAMP: cyclic adenosine monophosphate
PKA: protein kinase A
PDE: phosphodiesterase
Ct: cycle treshold
Ni–NTA agarose: nickel-nitrilotriacetic acid agarose
RIPA: radioimmunoprecipitation assay
AMPK: AMP-activated protein kinase
